# Melatonin Metabolism in the Central Nervous System

**DOI:** 10.2174/157015910792246244

**Published:** 2010-09

**Authors:** Rüdiger Hardeland

**Affiliations:** Johann Friedrich Blumenbach Institute of Zoology and Anthropology, University of Goettingen, Goettingen, Germany

**Keywords:** Kynuramines, melatonin, 5-methoxytryptamine, N-acetylserotonin, reactive nitrogen species, reactive oxygen species, 6-sulfatoxymelatonin.

## Abstract

The metabolism of melatonin in the central nervous system is of interest for several reasons. Melatonin enters the brain either *via *the pineal recess or by uptake from the blood. It has been assumed to be also formed in some brain areas. Neuroprotection by melatonin has been demonstrated in numerous model systems, and various attempts have been undertaken to counteract neurodegeneration by melatonin treatment. Several concurrent pathways lead to different products. Cytochrome P_450_ subforms have been demonstrated in the brain. They either demethylate melatonin to *N*-acetylserotonin, or produce 6-hydroxymelatonin, which is mostly sulfated already in the CNS. Melatonin is deacetylated, at least in pineal gland and retina, to 5-methoxytryptamine. *N*^1^-acetyl-*N*^2^-formyl-5-methoxykynuramine is formed by pyrrole-ring cleavage, by myeloperoxidase, indoleamine 2,3-dioxygenase and various non-enzymatic oxidants. Its product, *N*^1^-acetyl-5-methoxykynuramine, is of interest as a scavenger of reactive oxygen and nitrogen species, mitochondrial modulator, downregulator of cyclooxygenase-2, inhibitor of cyclooxygenase, neuronal and inducible NO synthases. Contrary to other nitrosated aromates, the nitrosated kynuramine metabolite, 3-acetamidomethyl-6-methoxycinnolinone, does not re-donate NO. Various other products are formed from melatonin and its metabolites by interaction with reactive oxygen and nitrogen species. The relative contribution of the various pathways to melatonin catabolism seems to be influenced by microglia activation, oxidative stress and brain levels of melatonin, which may be strongly changed in experiments on neuroprotection. Many of the melatonin metabolites, which may appear in elevated concentrations after melatonin administration, possess biological or pharmacological properties, including *N*-acetylserotonin, 5-methoxytryptamine and some of its derivatives, and especially the 5-methoxylated kynuramines.

## INTRODUCTION

**Fig. (1):**
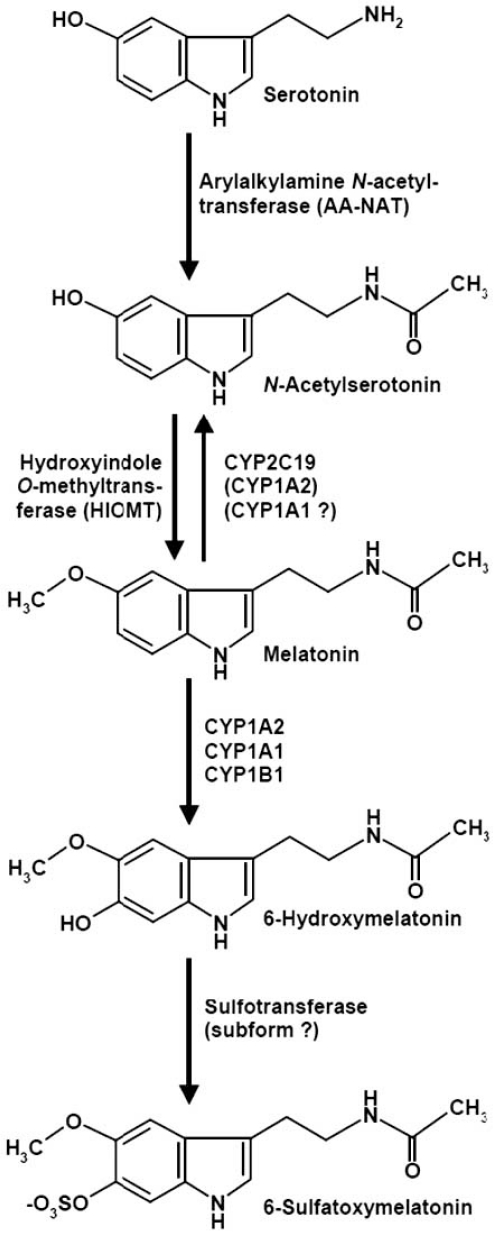
Synthesis and CYP metabolism of melatonin in the CNS. CYP = cytochrome P_450_.

Melatonin (*N*-acetyl-5-methoxytryptamine; Fig. **1**) had been first identified as the hormone of the pineal gland. Meanwhile, numerous additional sites of formation and a multitude of functions have been identified, which exceed the first-discovered roles of this indoleamine as a melanosome-concentrating agent in fish and amphibia and a mediator of the signal “darkness“ in the vast majority of vertebrates [39, 47, 137]. Considerable differences exist between melatonin formed in the pineal gland and in other organs, especially with regard to tissue retention, local metabolism and release to the circulation. In mammals, the contribution of extrapineal sources to circulating melatonin is usually low [39, 47, 55], mainly with the exception of the gastrointestinal tract, from which the indoleamine is released at substantial concentrations, but only under the influence of nutritional factors [57], in terms of a postprandial response [17, 44]. The amounts of gut-derived melatonin which appear in the circulation can acutely exceed by far those secreted by the pineal [57] and may lead to central nervous actions, but their chronobiological effects remain weak, for reasons related to the phase-response curve for melatonin [44].

By virtue of its amphiphilicity, melatonin can easily cross the blood-brain barrier [107, 109]. It can enter the CNS and the cerebrospinal fluid (CSF) *via *the choroid plexus [74]. This should be particularly important after administration of exogenous melatonin. To what extent the circulating hormone contributes, under physiological conditions, to its brain concentrations is not fully understood, specially as the pineal itself releases the indoleamine at much higher concentrations directly *via *the pineal recess to the third ventricle [146-148]. The appearance of melatonin in the third ventricle has been very recently demonstrated in humans, but the amounts reported are relatively moderate (about 8.75 pg/mL) [74]. Even lower concentrations were measured in the lateral ventricles. In a study comparing melatonin levels in mouse serum and cerebral cortex, the cortical concentrations amounted to 1% or less of those found in serum [70]. Approximately the same ratio was found after administration of exogenous melatonin. One should, however, be always aware that concentrations tell little about amounts taken up, since a compound may readily disappear if it is rapidly metabolized. In fact, the same study reported for the cerebral cortex concentrations of the metabolite 6-sulfatoxymelatonin by about 3 orders of magnitude higher than those of melatonin [70].

It seems possible that considerable regional differences exist within the brain. Some areas have been suspected to also synthesize melatonin, as discussed elsewhere [47]. However, this is still a matter of great uncertainties. In conjunction with studies on the acoustic tract of guinea pigs, melatonin was not only detected in the membranous cochlea, but also in the cochlear nerve [14]. Melatonin was reported to be released from the hypothalamus [161], a site at which high nocturnal levels had been found in an earlier study [94]. The rate-limiting enzyme of melatonin biosynthesis, arylalkylamine *N*-acetyltransferase (AA-NAT), is expressed in various parts of the central nervous system, such as cerebellum, olfactory bulb, prefrontal cortex, hippocampus and striatum, but it has remained unclear whether the product, *N*-acetylserotonin (NAS), is really converted to melatonin or acts independently as a neurotransmitter with functions of its own [46, 151, 152]. Elevated levels of melatonin were described for nucleus gracilis, pons [116], medulla oblongata [94, 116] and cerebellum [69, 94]. Data on brain levels of melatonin are still highly divergent. In earlier publications, rather moderate concentrations were usually found in the brain areas mentioned. In B6C3F1 mice, melatonin was reported to attain levels of about 0.4 pM in the cerebral cortex [70], whereas whole brain concentrations of up to 0.7 µM were measured in another, more recent study in Tg2576 mice [85]. Confirmation of these data and clarification would be of utmost importance. Mouse strains can vary enormously with regard to their melatonin levels. Some if them are practically melatonin-deficient, others exhibit strongly reduced levels of the indoleamine. B6C3F1 mice are F1 hybrids between melatonin-deficient C57BL/6 and melatonin-proficient C3H mice. Determinations of melatonin and metabolites had been carried out during the circadian minimum, between 9:00 and 14:00, the serum levels were in the range between 0.07 and, in 6 months old animals, 0.3 nM [70]. It would be of interest to know which concentrations might be attained at night and to what extent the genotype had influenced the results.

The problem of whether high AA-NAT activities reflect high melatonin levels becomes particularly obvious in the retina. In numerous non-mammalian and mammalian species, melatonin is rhythmically synthesized in this specialized CNS structure, in both a subpopulation of photoreceptor cells and the retinal pigment epithelium. In some non-mammalian organisms, it is also released to the blood in certain quantities [19, 30, 35, 77, 145, 153, 155]. However, primates and ungulates so-far investigated exhibited high nocturnal expression levels of AA-NAT, but are reported to not form melatonin at substantial rates, because the NAS-converting enzyme, hydroxyindole *O*-methyltransferase (HIOMT) is poorly expressed [13, 65, 66]. However, recent data show HIOMT expression in cultured human retinal pigment cells (ARPE-19 cells) [162]. The absence of HIOMT does not seem be the case throughout the non-pineal areas of the CNS, since this enzyme or its mRNA were sometimes detected. Moreover, the enzyme may be partially replaced by other, non-specific *O*-methyltransferases [47].

Melatonin metabolism in the CNS is particularly relevant for several reasons. The relative contribution of concurrent catabolic pathways may be strongly influenced by conditions of inflammation and oxidative stress [50]. This is supported by findings on elevated levels of melatonin’s oxidation product N1-acetyl-N2-formyl-5-methoxykynuramine (AFMK) in the cerebrospinal fluid of patients with meningitis [125]. This metabolite and also its secondary product, N1-acetyl-5-methoxykynuramine (AMK), have been shown to possess cell-protective properties including beneficial effects in mitochondria [1, 40, 41, 50, 71, 136, 143]. Moreover, numerous publications have demonstrated the neuroprotective potential of melatonin under various experimental conditions [11, 85, 104, 105, 108, 118, 131, 154]. For this purpose, elevated doses of melatonin strongly exceeding physiological levels have to be used, especially under acute conditions requiring the prevention of neuronal dysfunction. Therefore, detailed informations on the fate of melatonin are indispensable if the compound shall be used either in animal models or for human treatment.

This article does not only intend to summarize the facts known to date, but should be also understood as an attempt to stimulate determinations of melatonin in the various brain areas and to follow up its metabolism under different conditions, including brain inflammatory diseases, neurodegenerative disorders and treatments with melatonin.

## CYTOCHROME P_450_ METABOLISM

Plasma melatonin is predominantly metabolized by hepatic cytochrome P450 enzymes (CYPs), mainly by the 6-hydroxylating subform CYP1A2. CYP1A1 and the extrahepatic CYP1B1 contribute to the formation of 6- hydroxymelatonin, whereas CYP2C19 and, to a much smaller extent, CYP1A2 can also demethylate melatonin to its precursor, NAS [79, 93]. Since the dealkylating activity of CYP1A1 is well-known for ethoxylated substrates [114, 156], NAS formation from melatonin seems likely for this isoenzyme, too. In addition to other CYPs not related to melatonin metabolism, CYP1A2 [60, 88, 126], CYP1A1 [60, 88], CYP1B1 [79, 88], and CYP2C19 [58, 59, 159] are also expressed in the brain. The presence of these CYP isoenzymes indicates that, at least, a certain fraction of melatonin should be either 6-hydroxylated or O-demethylated in the CNS (Fig. **1**). Direct information based on determinations of conversion rates and metabolite concentrations would be required to definitely judge the relevance of these routes. However, it may become difficult to distinguish NAS formation *via *AA-NAT from that *via *dealkylating CYPs. A possibility for testing this might be the use of CYP inhibitors. For instance, fluvoxamine, otherwise used for modulating serotonin reuptake, inhibits both CYP1A2 and CYP2C19 [8]. Another CYP2C19 inhibitor is the antifungal drug fluconazole [75]. With such compounds, changes in brain levels of melatonin and its metabolites may be detected.

In contrast to the presence of hydroxylating CYP isoforms in the CNS, a pioneering study in which the fate of intracisternally injected, radiolabeled melatonin was followed, no 6-hydroxymelatonin was detected among the products [52]. Instead, AFMK and AMK were discovered in that investigation and described as major brain metabolites of melatonin. The meaning of this finding will be discussed below in another section. The undetectable amounts of 6-hydroxymelatonin are well in accordance with very low levels of 6-hydroxymelatonin in the cerebral cortex of mice (0.1% of corresponding melatonin), as reported in a more recent study [70]. However, this recent investigation also showed amounts of 6-sulfatoxymelatonin which were by several orders of magnitude higher. This compound may not have been detected in the earlier pioneering study, already for reasons of extraction. Contrary to the mouse brain, CSF concentrations of 6-sulfatoxymelatonin in the human ventricles were reportedly lower than those of melatonin [74]. This confirms, at least, the appearance of the conjugated metabolite in the human brain, but determinations in the solid tissue may reveal different values.

If 6-hydroxymelatonin is formed in the brain and subsequently conjugated, the biological meaning of this pathway will have to be clarified. Hepatic 6-hydroxylation of melatonin is a prerequisite for subsequent conjugation and excretion as 6-sulfatoxymelatonin. The extremely higher cortical amounts reported for murine 6-sulfatoxymelatonin relative to those of 6-hydroxymelatonin [70] may indicate that either the conjugated metabolite is not easily released to the circulation because of its electrical charge or the compound plays an additional, to date unknown role in the CNS. A specific 6-hydroxymelatonin sulfotransferase has not yet been identified in the CNS. However, the brain is known to express various sulfotransferases. Although the focus has mostly been on sulfation of polysaccharides in the extracellular matrix, low molecular weight metabolites have also been shown to be conjugated by sulfotransferase subforms, including those catalyzing the sulfation of catecholamines [117], xenobiotics [133], *N*-acylated dopamine derivatives [4], thyroxine [133] and neurosteroids [31]. In the case of neurosteroid sulfates, specific neuromodulatory roles different from the non-conjugated molecules have been assumed [31]. Although it would be pure speculation to assume a separate physiological role for 6-sulfatoxymelatonin, this may be worthy of being studied, also with regard to the relatively high levels found in the mouse CNS.

Assumptions on eventual metabolic routes for eliminating 6-sulfatoxymelatonin could, again, be nothing more than speculation. If such pathways exist at all, a theoretical possibility might be sought in the deacetylation, especially by aryl acylamidases (AAAs) in the broadest sense, including a more specific melatonin deacetylase. These enzymes, which will be discussed in the next section, can deacetylate melatonin [49] and NAS [53] to 5-methoxytryptamine (5-MT) and serotonin (5-HT), respectively, thereby allowing a rapid further degradation of the deacetylated products by monoamine oxidase A (MAO A) to give the substituted indole-3-acetaldehydes, which are converted to the 5-methoxylated or -hydroxylated indole-3-acetic acids or substituted tryptophols [46]. Although various indolic compounds have been tested as AAA substrates or inhibitors, pertinent data on 6-hydroxymelatonin or 6-sulfatoxymelatonin are entirely missing.

## MELATONIN DEACETYLATION

**Fig. (2):**
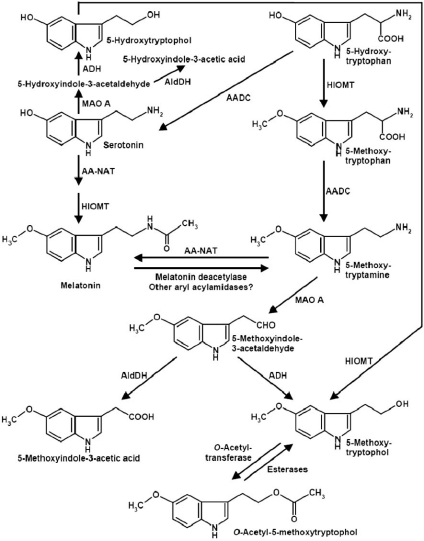
The complex network of methoxyindole metabolism in the CNS. AADC = aromatic amino acid decarboxylase; ADH = alcohol dehydrogenase; AldDH = aldehyde dehydrogenase; MAO = monoamine oxidase; other abbreviations as in Fig. (**1)**.

Melatonin deacetylation to 5-MT is observed in some areas of the CNS, but there seem to be considerable differences concerning species and sites. Moreover, the contribution of different enzymes and their subforms may not have been fully clarified. In general, melatonin deacetylating enzymes can be classified as aryl acylamidases (AAAs). Although the conversion of melatonin to 5-MT by these enzymes seems to be important in organisms different from animals, such as dinoflagellates [37, 45] and yeast [129], the quantitative significance of this pathway has only become apparent in the retinas of some amphibians, reptiles and fish [19, 32, 34]. In other retinas in which AA-NAT is strongly expressed, but only small amounts of melatonin are detected, melatonin deacetylation may be considered, too, as long as the low melatonin levels are not attributable to poor *O*-methylation. The occurrence of melatonin deacetylation in a broader range within the CNS cannot be ruled out. Apart from the fact that this pathway was also found in vegetative tissues, the respective enzyme activity was clearly demonstrated in the pineal gland and, in some reptiles, also in other brain regions [33] (Fig. **2**). The enzyme identified in the *Xenopus* retina was later named melatonin deacetylase [32-34]. It displays high substrate specificity for melatonin and is clearly distinct from different, less specific AAAs. AAAs from rat and bovine pineal glands [53] may represent forms of melatonin deacetylase. Its low pH optimum (about pH 5) is reminiscent of a previously described subform AAA-2, which was also detected in the rat brain [54]. The deacetylating properties of other AAAs turned out to represent side activities of acetylcholinesterase [6, 29, 91, 92], butyrylcholinesterase [6] or even human serum albumin [83]. It has remained uncertain to what extent non-specific AAAs of vertebrate origin are capable of deacetylating melatonin at all. 5-MT formation from melatonin was reported for mammalian liver, whereas, in the same studies, no such conversion was observed in the brain [9, 112].

For two fundamental reasons, it is impossible to conclude on melatonin deacetylation on the basis of 5-MT concentrations. Several earlier findings obtained *in vivo* or in brain slices may have to be revisited under this aspect. The first problem concerns the fast destruction of 5-MT by MAO A. Contrary to melatonin and NAS, the non-acetylated indoleamines in general and 5-MT in particular represent rapidly converted substrates of MAO A [28, 46]. Reliable 5-MT measurements in the pineal gland require the presence of MAO inhibitors [28, 101]. The second difficulty consists in the multiplicity of pathways leading to 5-MT [46] (Fig. **2**). Apart from melatonin deacetylation, this indoleamine can be also formed from the precursor 5-hydroxytryptophan (i) *via O*-methylation and subsequent decarboxylation of the resulting 5-methoxytryptophan, and (ii) by *O*-methylation of serotonin. As an additional complication, 5-MT was reported to be demethylated to serotonin by human CYP2D6, a subform present in the CNS [160]. However, an earlier study in which deuterated 5-MT was administered to rats did not support the quantitative relevance of this pathway, since only labeled 5-methoxyindole-3-acetic acid, but not 5-hydroxyindole-3-acetic acid were detected in the urine [73]. If this is not a matter of species differences between rats and humans, 5-MT demethylation may, thus, be only of interest in experiments using high pharmacological concentrations. 

In the pineal gland of Syrian hamsters, the contribution of melatonin to 5-MT formation seems to be marginal, compared to that of the other indoles. This can be concluded from the phase positions of the respective circadian rhythms. The rhythm of 5-MT exhibited a diurnal maximum, similar to that of serotonin, and was, thus, strongly out of phase with the nocturnally peaking melatonin rhythm [102]. Whether the same is valid for other species has not yet been investigated on a broader scale.

In the pineal gland, 5-MT, whether formed from melatonin, 5-methoxytryptophan or serotonin, is clearly catabolized by MAO A, and not MAO B [84, 103], although the pinealocytes only express MAO B [84]. MAO A was found to be localized only in the noradrenergic nerve endings, so that pineal-derived 5-MT may be locally catabolized in the noradrenergic compartment [84], whereas another fraction can be released from the gland to the circulation or, what remains to be studied, to the third ventricle, as is the case with melatonin. The product of amine oxidation, 5-methoxyindole-3-acetaldehyde, can be either converted by aldehyde dehydrogenase to an excreted end product, 5-methoxyindole-3-acetic acid, or by alcohol dehydrogenase to 5-methoxytryptophol, a compound that displays some biological activities [46]. Again, the two methoxylated metabolites are also formed by secondary *O*-methylation of the respective 5-hydroxyindoles [46].

5-MT and the metabolite 5-methoxytryptophol have been considered in the past as additional neurohormones and/or neuromodulators. Although various effects of these compounds have been described and although 5-MT is frequently used in pharmacological experiments [46], also concerning specific 5-HT receptor subforms, available data may not suffice for documenting physiological roles of these compounds in vertebrates. The same may be valid for the *O*-acetyl-5-methoxytryptophol, a compound present in the pineal gland and differing from melatonin only by the replacement of the aliphatic nitrogen by an oxygen [46, 128]. This almost forgotten metabolite (Fig. **2**) was reported to inhibit nicotinic and muscarinic acetylcholine receptors [26] and, in Syrian hamsters, to decrease pituitary prolactin and LH levels [76], but again, these effects are presumably only of pharmacological nature. This compound is very unstable in the presence of ubiquitously abundant esterases [72].

In conclusion, one can state that, among the vertebrate organs studied, a relevant rate of melatonin deacetylation is only demonstrated in the retinas of fish, amphibians and reptiles. In the pineals of various vertebrates, the pathway exists, too, but seems to be of minor importance. In vertebrates, the physiological relevance of 5-MT and its metabolites is still uncertain. It may be noted that this is not generally the case in the living world. In dinoflagellates, 5-MT is a much more powerful agent than melatonin [7, 45]. Beyond the specific melatonin deacetylases found in retinas and pineals, the role of other AAAs has remained unclear. Various of the earlier studies, including those conducted in brain tissue, describe inhibitions of AAA activity by various indoles, such as serotonin, NAS, and 5-MT, when measurements were carried out using artificial substrates [29, 54, 91, 92]. Unfortunately, these investigations did not consider melatonin. Inhibition by melatonin was, at that time, only reported for the pineal enzyme [53] and may, thus, concern the specific melatonin deacetylase. Competition with synthetic substrates can indicate either inhibition or binding as a substrate or product, what had not been clearly distinguished in those earlier studies. Hence, a role of melatonin as a ligand of unspecific brain AAAs may not be entirely ruled out, also with regard to the fact that a rapid decay by MAO A had previously not been taken into account. An eventual function of melatonin as a ligand could also be that of an inhibitor. This is presumably irrelevant at basal physiological levels, but might be considered after administration of exogenous melatonin. Reductions of acetylcholinesterase activity by melatonin have been observed in various mouse brain regions in vivo, under experimental conditions of scopolamine-induced amnesia [3], but these effects may have been of indirect nature. Hybrid molecules between melatonin and the established acetylcholinesterase blocker tacrine were reported to be more efficient inhibitors of this enzyme than tacrine, and to bind to both the catalytic and the peripheral anionic sites [25]. These findings may be seen as a reason for re-investigating possible interactions of melatonin with the acylcholinesterases. In this context, it may be briefly noted that the brain AAAs have recently, and somehow unexpectedly, re-gained some interest after they were shown to be inhibited by several investigational anti-Alzheimer drugs considered for human treatment [20, 22, 100]. With regard to the attempts of antagonizing Alzheimer’s disease by melatonin [85, 95, 130, 131], this possibility may be kept in mind, although melatonin exhibits numerous other properties of interest in this neurodegenerative disorder.

## NON-ENZYMATIC HYDROXYLATION AND NITROSATION

Owing to its radical scavenging properties [39, 41, 44, 47, 104, 106-109, 134, 137, 141, 142], melatonin can lead to several products by interacting with reactive oxygen and nitrogen species. Although various different oxidants can react with melatonin, the focus has frequently been on the products generated by hydroxyl radicals. Because of the non-enzymatic nature of these reactions, these pathways are independent of species (in aerobic organisms), tissue, cell type and compartment, whereas their rates may be strongly influenced by the highly variable local abundance of these radicals. The same should be valid for nitrosation of melatonin, in all NO-synthesizing organisms. Under basal physiological conditions, the formation of radical-generated products is presumably very low and may be overlooked when following the quantitative entrance into concurrent pathways. However, under conditions of oxidative stress and, in particular, at sites of inflammation or, even more, in systemic sepsis, the products can become detectable and eventually relevant. This is especially the case when melatonin is administered at high concentrations under experimental conditions designed to counteract an artificially induced oxidative or nitrosative stress. Since various of the non-enzymatic products are also biologically active and undergo redox reactions [41, 44, 47, 141], these metabolites should not be neglected in experiments on protection by melatonin.

**Fig. (3):**
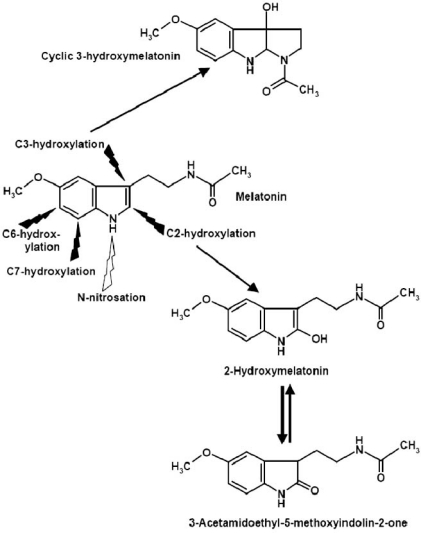
The preferred sites of non-enzymatic hydroxylation and nitrosation at the melatonin molecule. Black flashes: hydroxylation; white flash: nitrosation.

Oxidation of melatonin by hydroxyl radicals leads to several hydroxylated products [142]. This type of conversion can be explained by interaction of melatonin with two hydroxyl radicals, one acting by hydrogen abstraction, the other by combining with the reaction partner. Hydroxylation can take place at various sites of the molecule, in particular, at C-atoms 2, 3, 6, and 7 [142] (Fig. **3**). *In vivo*, 6-hydroxy-melatonin formed this way may not be easily distinguished from the enzymatically produced, more abundant fraction. 2-Hydroxymelatonin, which has been repeatedly detected under experimental conditions, is in equilibrium with its tautomer, 3-acetamidoethyl-5-methoxyindolin-2-one (= “2-oxomelatonin“) [2, 39, 41] (Fig. **3**). 7-Hydroxymelatonin has been rarely considered, although the calculated activation energy for the respective reaction is as low as that for 6-hydroxylation [142]. 3-Hydroxylation leads to an unusual compound, cyclic 3-hydroxymelatonin (c3OHM) [139]. The formation of this molecule demonstrates the relevance of the aliphatic side chain for the redox properties of melatonin, although this was originally not foreseeable. c3OHM was detected in the urine of rats and humans [138, 139]. Its concentration increased considerably after exposure of rats to ionizing radiation [139]. Therefore, c3OHM may be regarded as a marker of oxidative stress, especially as far as this is related to elevated generation of hydroxyl radicals. c3OHM may be also formed upon interaction with other free radicals, but the chemical mechanisms are poorly understood. To date, the production of c3OHM in the absence of hydroxyl radicals has been convincingly demonstrated with a synthetic, low-reactivity radical, the ABTS cation radical [ABTS = 2,2´-azino-*bis*-(3-ethylbenzthiazoline-6-sulfonic acid)] [135]. In mice, c3OHM was especially detected in the urine after administration of exogenous melatonin [135, 138]. However, the major fraction of c3OHM was reported to be present as a sulfate conjugate [80]. The presence of c3OHM sulfate in mouse urine contrasts to the finding that, in this species, most of the 6-hydroxymelatonin appeared as glucuronide, not as sulfate [78]. Differences in conjugation may exist between vegetative and central nervous conjugation processes, but this remains to be investigated in detail.

The chemical structure of c3OHM is insofar of special interest as is reveals remarkable homology to well-known acylcholinesterase inhibitors, such as eserine (physostigmine). On this basis, several derivatives of c3OHM have been synthesized and investigated for inhibition of acetyl- and butyrylcholinesterase activities [127]. This might be of value with regard to the assumed possibility of interfering with the progression of Alzheimer’s disease by inhibiting the acylcholinesterases. The unsubstituted c3OHM which lacks a free aliphatic *N*-acetyl group is obviously no specific inhibitor of these enzymes. Data on melatonin and other *N*-actylated analogs have not been disclosed.

Melatonin *N*-nitrosation represents another type of non-enzymatic metabolism that has been repeatedly investigated. Formation of 1-nitrosomelatonin (= *N*-nitrosomelatonin) was observed with various NO donors [15, 96, 149, 150] and also with peroxynitrite [15, 96]. In the presence of NO, melatonin nitrosation was reported to be promoted by NO_2_, an effect interpreted in terms of sequential reactions with the two reactive nitrogen species [96]. Alternately, a reaction with N_2_O_3_ might be considered as well, since this molecule represents an easily formed adduct of these two nitrogen species and is known to be a potent nitrosating agent [48,50].

1-Nitrosomelatonin may be relevant to the CNS, with regard to the role of NO in neuronal excitation and, also, to the possibility of microglia activation. This might be especially the case under experimental conditions of melatonin administration in models of excitotoxicity, oxidotoxicity, or brain inflammation. The pharmacokinetics of 1-nitroso-melatonin in the brain has been studied [97]. The role of 1-nitrosomelatonin strongly differs from those of the melatonin metabolites formed by interaction with reactive oxygen species, and the nitrosation of this indole should not be misinterpreted in terms of detoxification. 1-Nitrosomelatonin easily re-donates NO [12, 16, 23] and is likewise capable of transnitrosating other molecules [63, 64]. These properties can be either desirable or highly undesirable. On the one hand, 1-nitrosomelatonin may be used as an amphiphilic NO source, and the idea has been that melatonin regenerated by NO release detoxifies the oxygen radicals formed as a secondary consequence of NO metabolism [12, 64]. On the other hand, transnitrosation of especially mitochondrial proteins can cause dysfunction of the respiratory chain and electron leakage [40, 48]. Under basal physiological conditions, 1-nitrosomelatonin is presumably formed at low rates, so that this aspect of melatonin metabolism should be only relevant in experimental model systems.

## THE KYNURAMINE PATHWAY

**Fig. (4):**
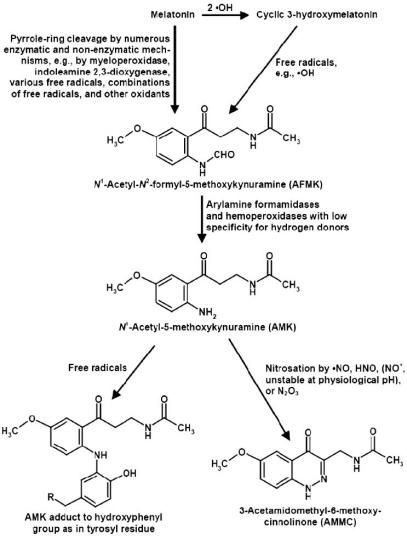
The kynuramine pathway of melatonin and some recently discovered products. For more details on the formation of AFMK see refs [38, 50, 93, 136], of AMK refs. [47, 50], of AMMC ref. [42], and of AMK adduct ref [89].

The melatonin-derived kynuramines AFMK and AMK (Fig. **4**) were discovered in a pioneering study [52], in which these compounds attained about one third of the products and were, thus, classified as major brain metabolites. Although pyrrole-ring cleavage represents a classic pathway of tryptophan catabolism, this is not generally the case with the oxidation of other indoles. That investigation demonstrated for the first time the formation of kynuramines from an indoleamine , whereas non-acetylated compounds like serotononin are predominantly catabolized by MAO A, as discussed above, and 5-hydroxylated indoles, such as serotonin and NAS frequently form dimers in non-enzymatic redox reactions [10, 56]. 

The occurrence and biological activities of kynuramines and, in particular, AFMK and AMK have been recently reviewed in detail [50]. In brief, AFMK is formed *via *pyrrole ring cleavage of melatonin by various catalysts, enzymes such as indoleamine 2,3-dioxygenase, myeloperoxidase, some other hemoperoxidases, by cytochrome c, various pseudoenzymatic catalysts, and in various reactions with reactive oxygen species, including free radicals and singlet oxygen [38, 39, 47, 50, 136, 141]. AFMK can also derive, *via *radical reactions, from c3OHM [135]. This multiplicity of pathways which lead to the same metabolite provokes the question of their relative contribution *in vivo*, especially in the CNS. Moreover, the relative amounts of AFMK and secondary products thereof, compared to other catabolic routes, is of interest. In the course of the original discovery of AFMK and AMK [52], the large melatonin fraction converted to these kynuramines of about one third was indicative of quantitative relevance.

Meanwhile, some doubts have arisen as to whether these high amounts are generally present in the brain. The original publication [52] is certainly reliable, since otherwise AFMK and AMK would not have been discovered. The question is, however, whether the entrance of such a high melatonin fraction into the kynuramine pathway might be conditional. This starts with the possibility that a much smaller proportion may be converted to kynuramines in the absence of external melatonin. Moreover, the route of administration may be of importance. In particular, an eventual microglia activation by high levels of melatonin might be considered. Surprisingly, little is known about the effects of melatonin on otherwise unchallenged microglia, whereas the counteraction of proinflammatory treatments and signals has been repeatedly studied. Some investigators concluded that melatonin does not exert relevant effects on resting microglia [121]. However, related cells such as macrophages, peripheral monocytes , cultured monocyte-derived cell lines and also postnatal microglia were reported to be activated by melatonin [21, 61, 87]. In monocytes, the melatonin effects were relatively short-acting and mainly concerned intracellular rises in reactive oxygen species [98, 99]. These effects were only observed at strongly elevated levels of melatonin and assumed to be mediated by calmodulin [98, 99], a protein capable of binding melatonin with low affinity [39, 47]. The response may be limited because of other actions by melatonin and its oxidatively formed metabolites. In peripheral monocytes, melatonin and, even more, AFMK suppressed TNF-α and IL-8 production [124] and, in macrophages, cyclooxygenase-2 and iNOS expression [24, 86]. Moreover, melatonin was found to be efficiently oxidized to AFMK by macrophages [124]. It remains to be studied whether microglia responds in a similar way to melatonin and AFMK. Activated microglia could convert melatonin by two enzymes, indoleamine 2,3-dioxygenase, although its main substrate is tryptophan, not melatonin [50, 51, 122, 132], and myeloperoxidase [27, 50, 111, 123, 157, 158]. Additionally, melatonin might be oxidized to AFMK by reactive oxygen species [38, 39, 47, 50, 136, 141] transiently formed in excess. Therefore, a scenario for elevated formation of 5-methoxykynuramines upon intracisternal melatonin administration [52] might include some transient effect on microglia, which may, thereafter, be stopped by AFMK. This effect may be only seen at elevated melatonin levels sufficient for actions *via *calmodulin, but this remains to be clarified. In such a case, melatonin administered *via *the drinking water or i.p. injections may not attain concentrations sufficient for microglia activation.

These considerations are not entirely hypothetical, since rises in CSF concentrations of AFMK were, in fact, observed under brain inflammatory conditions. In the cerebrospinal fluid of patients with viral meningitis, elevated levels of AFMK were detected, in conjunction with a negative correlation to some interleukins [125]. In CSF samples containing more than 50 nM AFMK, protein concentrations and levels of IL-8 and IL-1β were much below those from persons with AFMK contents between 10 and 50 nM. The relationship to the cytokines indicates an interconnection to the immune system, but oxidative stress because of the inflammation may have contributed to melatonin oxidation. These findings demonstrate that AFMK formation is of pathophysiological interest, including its possible usefulness as an indicator molecule [50].

Also beyond its antiinflammatory actions, AFMK has been repeatedly shown to protect against oxido- and excitotoxicity [18, 50, 81, 82, 90, 136]. These effects included protection against radiation [81, 82] and prevention of radiation-induced inhibition of neurogenesis and memory impairment [82]. In terms of direct antioxidant actions, the capability of AFMK of detoxifying hydroxyl radicals seems plausible, because of the particularly high reactivity of this oxygen species. However, AFMK is generally less reactive than its precursor, melatonin, and its secondary product, AMK [38, 50, 110]. Because of its preference for two-electron transfer reactions, as demonstrated by cyclic voltammetry [136], interactions with free radicals are not favored, compared to other redox reactions [38]. This might indicate the existence of some signaling properties of AFMK, which have, however, not yet been identified, although additional hints for this exist from other experiments not designed for protection [50]. Nevertheless, AFMK does interact with free radicals of low reactivity, as far as they possess a sufficiently long life-time. With ABTS cation radicals, several previously unknown products were obtained, such as *N*-(1-formyl-2-hydroxy-5-methoxy-3-oxo-2,3-dihydro-1H-indol-2-ylmethyl)-acetamide, E- and Z-isomers of *N*-(1-formyl-5-methoxy-3-oxo-2,3-dihydro-1H-indol-2-ylidenemethyl)-acetamide, as well as some deformylated analogs [113]. These reactions should also be possible with other resonance-stabilized organic radicals formed in biological material, but to date it is unknown whether the discovered C2-substituted 3-indolinones is physiologically relevant or only a matter of chemistry.

AFMK can be deformylated to AMK by, at least, three different mechanisms. One of them, a photochemical reaction [120], should be irrelevant to the brain, but may occur in the eye. Another, long-known reaction is catalyzed by aryl-amine formamidases, a group of enzymes, some of which have a relatively low substrate specificity [49, 50, 62]. The conversion of AFMK to AMK was demonstrated in the brain [62]. Other enzymes capable of deformylating AFMK are hemoperoxidases with low specificity for hydrogen donors, including the peroxidase activity of catalase [140]. As a consequence of hydrogen donation, the resulting imino intermediate is hydrated and the carbamate thereby formed releases CO_2_ [47, 50]. 

Once produced, AMK can rapidly disappear, because of numerous reactions it can undergo with various reactive oxygen and nitrogen species. The transitory nature of this compound has been particularly addressed in a recent review paper [50]. Therefore, difficulties in detection should not be immediately misinterpreted as a lack of formation. However, physiological levels of AMK in the brain are still unknown. AMK is of high interest because of several properties. It is a potent antioxidant [110], effective scavenger of reactive nitrogen species [36, 42, 43], protecting agent against mitochondrial damage [1, 38, 40, 41], downregulator of cyclooxygenase-2 [24, 86], cyclooxygenase inhibitor by far more potent than aspirin [62], and antagonist of neuronal [71] and inducible NO synthases [24, 143].

Products formed from AMK by interaction with oxidizing free radicals are only partially known. In chemical systems, several dimers and oligomers have been identified [144], which are, however, presumably physiologically irrelevant, because of much lower educt concentrations present in the biological material. It seems more likely that AMK intermediates formed by oxidants rather interact with other aromates, which has, in fact been demonstrated with tyrosine [50, 89] and tryptophan [50]. To date, it is unclear whether or not such reactions represent undesirable or beneficial actions. On the one hand, unspecific binding to aromatic residues of protein side chains may cause dysfunction or, perhaps, be immunogenic [89], but AMKylation of proteins otherwise regulated by tyrosine phosphorylation may be prevented from activation, which can be of advantage when cell division is unfavorable, such as in neoplasmic tissue [50]. Other products formed by interaction with highly reactive oxidants, such as hydroxyl radicals [50], carbonate radicals [110] and singlet oxygen [119] have led to substantial destruction of the AMK molecule, up to a decomposition of the aromatic moiety [50].

Among the products formed by interaction with reactive nitrogen species, three main products were obtained in chemical systems, *N*^1^-acetyl-5-methoxy-3-nitrokynuramine (AMNK, 3-nitro-AMK), *N*-[2-1] acetamide (MQA) and 3-acetamidomethyl-6-methoxy-cinnolinone (AMMC) [36, 68] (Fig. **4**). AMNK was formed by interaction with the peroxynitrite-CO_2_ adduct (ONOOCO_2_^–^), which decomposes to the carbonate radical, (CO_3_**·**^–^) and **·**NO_2_, a physiological nitration mixture. This reaction should be possible at any site in the organism at which AMK encounters ONOOCO_2_^–^, including the CNS. The second compound, MQA, has meanwhile been detected as a metabolite of yeast, after incubation with AFMK [67], but to date no indication exists for its formation in brain. However, the third metabolite, AMMC, deserves more attention with regard to AMK metabolism in the CNS, because of the relatively high rates of NO generated in this organ. AMK is readily formed with any nitrosating agent, in particular, all NO congeners, NO^+^, **·**NO and HNO, the protonated NO^–^ subform present at physiological pH, and N_2_O_3_ as well [42, 50]. For the different mechanisms of reactions with NO subforms see ref. [42]. Additional routes by physiologically present transnitrosating agents can be expected. A remarkable difference exists between AMK and other compounds as NO scavengers. Typically, nitrosated products re-donate NO, as has been also described for 1-nitrosomelatonin [12, 16, 23], they transnitrosate other molecules [63] or decompose as nitrosamine intermediates to diazonium ions, which may either lead to toxic and mutagenic carbenium ions or, in *o*-hydroxylated compounds, to oxadiazoles and their tautomers, *o*-quinone diazides [5]. This last pathway has been reported for other tryptophan metabolites, 3-hydroxyky-nurenine and 3-hydroxyanthranilic acid. However, *N*-nitrosation of AMK leads, by formation of a second ring, to the stable compound AMMC, which does not spontaneously re-donate NO [43].

As far as AMK is formed in the CNS, it will likely be converted to AMMC. To date, brain concentrations of AMMC, AMNK and MQA are unkown. Considerable differences can be expected with regard to the appearance of the precursor AFMK. This compound may be increased upon melatonin administration and, according to the CSF data mentioned [125], especially under conditions of brain inflammation. Levels of the novel metabolites formed from AFMK may also be of interest from a pathophysiological point of view.

## CONCLUSION

Although melatonin has been discovered more than half a century ago and although countless publications have dealt with the neuroprotective actions of melatonin, surprisingly little is known about brain concentrations of this indoleamine under basal physiological conditions. To some extent, part of the problem may be the use of different mouse strains, which vary considerably with regard to their capability of producing melatonin. It is recommended to perform urgently needed advanced studies on brain melatonin either in strains known to synthesize and secrete melatonin at nocturnal levels comparable to those known from other mammals, and/or to use other species. 

Even less can be said with certainty about the levels of its metabolites in the CNS and on the rates at which melatonin is converted by the various concurrent catabolic pathways. All of the pathways identified to date are of considerable interest, for different reasons. First, the fate of melatonin has to be known, which enters the brain *via *the pineal recess or the choroid plexus, or which may be synthesized within the brain. Second, the amounts of the respective metabolites have to be identified because of their additional biological actions.

According to recent findings, the main route of melatonin catabolism in the CNS may be that of hydroxylation and sulfation, although an immediate release of 6-sulfatoxy-melatonin from the brain, for purposes of excretion, is not very likely. Relatively high amounts of the conjugate described in, at least, one study [70] might indicate its extended persistence in the CNS. It will be an intriguing question whether 6-sulfatoxymelatonin may exert neuromodulatory effects, as described for other sulfated compounds formed in the CNS.

Demethylation to NAS and deacetylation to 5-MT are other pathways which deserve attention. Since NAS is also synthesized in some brain regions without further transformation to melatonin, the quantities obtained from melatonin demethylation may be of minor relevance, relative to direct NAS synthesis from serotonin. The role of 5-MT is still somehow enigmatic. Determinations of 5-MT in the absence of MAO A inhibitors may be highly misleading. Again, judgements are difficult because of 5-MT synthesis *via *other routes. The importance of melatonin deacetylation is best understood in the retinas of non-mammalian species, but this route should be re-considered in studies on melatonin metabolism in mammalian eyes. Moreover, a possible role of melatonin in acylcholinesterases, otherwise known to possess aryl acylamidase activities, seems worthy to be investigated in depth. A particular reason for this suggestion is derived from the inhibition of these enzymes by both other indoleamines and several investigative Alzheimer drugs. 

The kynuramine pathway, originally considered as a major route of melatonin metabolism in the brain, should be re-investigated with regard to its quantitative importance. This is necessary because bioactive and neuroprotective compounds, AFMK and AMK, are formed in this pathway. A remarkable spectrum of effects is known especially for AMK, which acts as an effective scavenger of reactive oxygen and nitrogen species, mitochondrial modulator, downregulator of cyclooxygenase-2, inhibitor of cyclooxygenase, neuronal and inducible NO synthases. Products deriving from AFMK and AMK have been identified, mostly in chemical systems, but their rates of formation in the brain remain to be determined. AFMK is synthesized by both enzymatic and non-enzymatic mechanisms, and all these routes are part of oxidative metabolism, either in relation to enzymes upregulated in activated microglia or to excessive generation of free radicals by whatever cell type. In the future, the relative rates of melatonin catabolism will have to be determined also under conditions of oxidative stress and, in particular, brain inflammatory diseases. Elevated AFMK levels in the human CSF of patients with meningitis have already been reported [125]. Following microglia activation, the kynuramine pathway may turn out to gain a quantitative relevance which substantially exceeds that under basal conditions. The same may be valid for other compounds formed by interaction with reactive oxygen and nitrogen species.
